# Post-diagnostic prescriptions for low-dose aspirin and breast cancer-specific survival: a nested case-control study in a breast cancer cohort from the UK Clinical Practice Research Datalink

**DOI:** 10.1186/bcr3638

**Published:** 2014-04-04

**Authors:** Liam J Murray, Janine A Cooper, Carmel M Hughes, Des G Powe, Chris R Cardwell

**Affiliations:** 1 Cancer Epidemiology and Health Services Research Group, Centre for Public Health, Queen’s University Belfast, Institute of Clinical Sciences Block B, Belfast, UK; 2 Centre of Excellence for Public Health (NI), Queen’s University Belfast, Institute of Clinical Sciences Block B, Belfast, UK; 3 School of Pharmacy, Queen’s University Belfast, Belfast, UK; 4 Department of Cellular Pathology, Queen’s Medical Centre, Nottingham University Hospitals NHS, Trust, Nottingham, UK; 5 The John van Geest Cancer Research Centre, Nottingham Trent University, Clifton Lane, Nottingham, UK

## Abstract

**Introduction:**

Recent observational studies indicate that post-diagnostic use of aspirin in breast cancer patients may protect against cancer progression perhaps by inhibiting cyclooxygenase-2 dependent mechanisms. Evidence also supports a crucial role for interactions between tumour cells and circulating platelets in cancer growth and dissemination, therefore, use of low-dose aspirin may reduce the risk of death from cancer in breast cancer patients.

**Methods:**

A cohort of newly diagnosed breast cancer patients (1998 to 2006) were identified in the UK Clinical Practice Research Datalink (and confirmed by cancer registry linkage). Cancer-specific deaths were identified up to 2011 from Office for National Statistics mortality data. A nested case-control analysis was conducted using conditional logistic regression to compare post-diagnostic aspirin exposure using General Practice prescription data in 1,435 cases (breast cancer deaths) with 5,697 controls (matched by age and year of diagnosis).

**Results:**

After breast cancer diagnosis, 18.3% of cancer-specific deaths and 18.5% of matched controls received at least one prescription for low-dose aspirin, corresponding to an odds ratio (OR) of 0.98 (95% CI 0.83, 1.15). Adjustment for potential confounders (including stage and grade) had little impact on this estimate. No dose response relationship was observed when the number of tablets was investigated and no associations were seen when analyses were stratified by receipt of prescriptions for aspirin in the pre-diagnostic period, by stage at diagnosis or by receipt of prescriptions for hormone therapy.

**Conclusions:**

Overall, in this large population-based cohort of breast cancer patients, there was little evidence of an association between receipt of post-diagnostic prescriptions for low-dose aspirin and breast cancer-specific death. However, information was not available on medication compliance or over-the-counter use of aspirin, which may have contributed to the null findings.

## Introduction

Evidence is accumulating that aspirin may protect against the development of some cancers, including breast cancer; for example, meta-analyses of observational studies indicate that breast cancer risk is reduced by 10 to 15% among aspirin users [1,2]. Recent evidence also points to a possible protective effect of aspirin against cancer progression in breast cancer patients. In the Iowa Women’s Health Study, the risk of death from breast cancer in postmenopausal breast cancer patients was reduced by approximately 50% among post-diagnostic users of aspirin and non-aspirin non-steroidal anti-inflammatory drugs (NSAIDs) [3]. Within the Nurses’ Health Study, similar reductions in the risk of distant recurrence and cancer-specific death were seen for breast cancer patients using aspirin after diagnosis [4,5]. However, in the Life After Cancer Epidemiology (LACE) study, post-diagnostic use of aspirin was not associated with risk of breast cancer recurrence, while users of ibuprofen had a substantial reduction in risk [6]. Each of these studies included patients with early stage disease (predominantly stage I and II), were undertaken within the United States (US), aspirin exposure was obtained by questionnaire and information was not available on the dose of aspirin used. Use of aspirin or non-aspirin NSAIDs at anti-inflammatory/analgesic doses may affect cancer progression by inhibiting prostaglandin endoperoxide synthase-2 (PTSG-2, cyclooxygenase-2) dependent mechanisms involved in cancer cell proliferation, motility, invasion and angiogenesis [7,8]. However, a growing body of evidence also supports a crucial but complex role for interactions between tumour cells and circulating platelets in cancer growth and dissemination [9,10]. It is therefore possible that the antiplatelet activity of (low-dose) aspirin may reduce the risk of metastasis in cancer patients by, for example, preventing angiogenesis or tumour cell extravasation and tissue invasion [10,11].

We investigated the association between post-diagnostic aspirin exposure and breast cancer-specific mortality in a large cancer registry defined population-based cohort of breast cancer patients in the United Kingdom (UK), in whom aspirin exposure was determined from prescription records. High dose aspirin is infrequently used in the UK and the primary focus of this study was exposure to low (antiplatelet) doses of aspirin.

## Methods

### Study design

A cohort study was conducted utilising recent linkages between the English National Cancer Data Repository (NCDR), the UK Clinical Practice Research Datalink (CPRD) and the Office of National Statistics (ONS) death registrations. The NCDR contains data on all cancer patients identified in English cancer registries, including date and site of primary cancer diagnosis, stage and treatment data. The CPRD is the world’s largest database of longitudinal patient records comprising around 6% of the UK population and includes demographic information, clinical diagnoses and prescription data which are of documented high quality [12]. Ethical approval for all observational research using CPRD data has been obtained by the CPRD Group from a Multicentre Research Ethics Committee (MREC). As the study had no direct patient involvement, patient consent for the study was not required. CPRD also contains ONS mortality data providing date and cause of death for deaths up to 2011.

Breast cancer patients were included if they had a CPRD breast cancer diagnosis code and a NCDR diagnosis code of primary breast cancer (based upon a relevant International Classification of Diseases (ICD) code C50.0 to C50.9 (version 10) or 174.0 to 174.9 (version 9)) from 1998 to 2007. Breast cancer patients with a previous NCDR cancer diagnosis, apart from *in situ* neoplasms and non-melanoma skin cancers, were excluded. The breast cancer cohort was linked to the CPRD (and ONS) data using a deterministic algorithm based upon the patient’s NHS number, gender, date of birth and postcode. After linkage, patients were excluded if they were prescribed hormone therapy (including tamoxifen and aromatase inhibitors) from general practice (GP) records more than eight weeks prior to their cancer diagnosis, indicating an incorrect breast cancer diagnosis date.

### Exposure data

Aspirin prescriptions (from GP data) were classified as low-dose if the drug strength was 75 mg or less (1.1% of prescriptions after cancer diagnosis were 25 mg, 97.1% 75 mg, and 1.8% 300 mg or higher). Duration of use was determined from the quantity of tablets prescribed. A quantity of 28 tablets, based upon the average, was assumed for less than 1% of prescriptions where quantity was missing or assumed incorrect.

### Covariates

Data available from NCDR included cancer stage, and chemotherapy and radiotherapy in the six months after diagnosis. Smoking, alcohol and body mass index (BMI) were determined from the closest GP record prior to breast cancer diagnosis (values older than 10 years were ignored). GP prescribing data were used to determine hormone therapy after cancer diagnosis (British National Formulary (BNF) chapter 8.3.4.1, including tamoxifen and aromatase inhibitors) and hormone replacement therapy (HRT) for oestrogen and progestogens (BNF chapters 6.4.1. and 6.4.2.) prior to diagnosis. GP prescribing data were also used to determine other medication exposure (statins, angiotensin converting enzyme (ACE) inhibitors, angiotensin receptor blockers (ARBs), beta-blockers and metformin). Comorbidities, prior to and during the relevant exposure period, were determined from GP diagnosis codes using a recent adaptation of the Charlson comorbidity index [13].

### Data analysis

The breast cancer cohort was initially analysed using a nested case-control approach, a common approach [14,15] which accounts for immortal time bias [16] without requiring complicated statistical techniques [17] and with minimal loss of precision [18]. Time varying covariate cohort analyses were also applied and presented as described later. Cases were members who had died due to breast cancer (on the basis of an ICD code for breast cancer as the underlying cause of death) and these were matched on age (in five year intervals) and year of cancer diagnosis (in one year intervals) to four controls who were alive after diagnosis for at least as long as their matched case. The exposure period in cases was the period from breast cancer diagnosis until six months prior to cancer-specific death. The exposure period in the controls was of the same duration as their matched cases starting from the date of breast cancer diagnosis. Prescriptions in the six-month period prior to death were removed as these may reflect end of life treatment or increased exposure to healthcare professionals (sensitivity analyses were also conducted excluding prescriptions in one year and two years prior to death). Consequently, the main analysis was restricted to individuals with at least one year of follow-up.

Conditional logistic regression was used to compare the risk of breast cancer-specific death by aspirin exposure (defined by number of tablets or prescriptions) calculating odds ratios (ORs) and 95% confidence intervals (95% CIs). Adjusted analyses were conducted including potential confounders, including stage, chemotherapy, radiotherapy, exposure to tamoxifen or aromatase inhibitors, comorbidities (including myocardial infarction, cerebrovascular disease, congestive heart disease, chronic pulmonary disease, peripheral vascular disease, peptic ulcer disease and diabetes) and pre-diagnosis smoking status. Similar analyses were conducted for all-cause mortality (where up to three controls were matched to each case). Various additional sensitivity analyses were conducted. Analyses were conducted excluding prescriptions in the first 12 months after cancer diagnosis as these may be influenced by cancer treatment. Analyses were conducted stratifying by aspirin exposure in the year prior to diagnosis and a separate analysis was conducted investigating aspirin prescriptions in the year prior to diagnosis. Analyses were also conducted by stage and adjusting for stage when restricted to registries with higher rates of available stage information. An analysis was also conducted in patients receiving prescriptions for hormone therapy (a proxy for oestrogen receptor positivity). All stratified analyses were conducted after re-matching cases to controls within the strata of interest. In another analysis, breast cancer-specific death was based upon a breast cancer ICD code recorded as any cause of death in ONS data, rather than just the underlying cause of death as used in the main analysis. Sensitivity analyses were also conducted analysing the entire breast cancer cohort, prior to conversion to case-control data, and applying survival analysis to investigate aspirin as a time varying covariate [16] (in which an individual was a non-user until first use and then remained a user until the end of follow-up, applying a six-month lag to mimic the case-control analysis). A separate analysis was conducted using this time varying covariate approach and also accounting for competing risk of deaths from other causes using the proportional subhazards model [19].

The final analysis contained 1,435 breast cancer-specific deaths and 5,697 matched controls, with low-dose aspirin usage of approximately 20%. This allowed 80% power to detect as significant at the 5% level an odds ratio of 0.80 in patients receiving low-dose aspirin equivalent to a 20% risk reduction in breast cancer-specific death. Statistical analyses were conducted in STATA 11 (StataCorp, College Station, TX, USA).

## Results

### Patient cohort

Overall, there were 11,863 primary breast cancers diagnosed between 1998 and 2007 identified in NCDR, with no previous record of cancer, that were linked to CPRD. Of these, we excluded 894 because the diagnosis date preceded CPRD research quality records, 116 due to unavailability of death registration data, 879 because they had less than one year of follow-up post diagnosis and 157 because hormone therapy records preceded the breast cancer diagnosis date by more than eight weeks. The final cohort for analysis therefore contained 9,817 breast cancer patients, with an average of 6.9 years of follow-up (range 1.0 years to 13.0 years) in those not dying, in whom there were 1,443 cancer-specific deaths. The breast cancer cohort was converted to nested case-control data with 1,435 cancer-specific deaths and 5,697 controls. No matched controls were available for eight cases and an additional eight cases had only three matched controls, seven had two matched controls and seven had one matched control.

### Patient characteristics

Table 1 shows characteristics of breast cancer-specific deaths (cases) and controls. The average time to death in the cases was 3.9 years and varied from 1 to 12.8 years. There was a greater proportion of higher stage cancers (*P* <0.001) and higher grade (*P* <0.001) in cases compared with controls and cases were more likely to have chemotherapy and less likely to have surgery within six months of diagnosis, compared with controls. Current smoking was more common in breast cancer-specific deaths compared with controls (21.0% versus 18.4%). Rates of comorbidities, alcohol consumption and BMI levels prior to diagnosis were generally fairly similar between cases and controls (Table 1).

**Table 1:** Characteristics of breast cancer patients who died from breast cancer (cases) compared with controls

	**Cancer-specific deaths n (%)**	**Controls n (%)**	***P*-value**
	**(n = 1,435)**	**(n = 5,697)**	
Year of cancer diagnosis			
1998 to 2000	500 (34.8%)	1,979 (34.7%)	Matched
2001 to 2003	518 (36.1%)	2,060 (36.2%)	
2003 to 2006	417 (29.1%)	1,658 (29.1%)	
Age at cancer diagnosis			
<40	92 (6.4%)	352 (6.2%)	Matched
40 to 49	247 (17.2%)	985 (17.3%)	
50 to 59	286 (19.9%)	1,144 (20.1%)	
60 to 69	273 (19.0%)	1,092 (19.2%)	
70 to 79	318 (22.2%)	1,271 (22.3%)	
80 to 89	181 (12.6%)	722 (12.7%)	
≥90	38 (2.7%)	131 (2.3%)	Matched
Follow-up time (years): *mean (sd)*	3.9 (2.3)	3.9 (2.3)	
*range*	1.0 to 12.8	1.0 to 12.8	
Grade			
Well	54 (5.9)	751 (19.4)	<0.001
Poor	377 (40.9)	1,900 (49.1)	
Moderate	492 (53.3)	1,217 (31.5)	
*Missing*	*512*	*1,829*	
Stage			
1	72 (11.0%)	1,133 (41.8%)	<0.001
2	402 (61.5%)	1,379 (50.8%)	
3	116 (17.7%)	167 (6.2%)	
4	64 (9.8%)	35 (1.3%)	
*Missing*	*781*	*2,983*	
Treatment within six months of cancer diagnosis			
Surgery	1,087 (75.8%)	4,842 (85.0%)	<0.001
Chemotherapy	573 (39.9%)	1,344 (23.6%)	<0.001
Radiotherapy	2,703 (47.5%)	700 (48.8%)	0.31
Smoking prior to cancer diagnosis			
Non-smoker	710 (59.9%)	3,037 (64.0%)	0.03
Ex-smoker	226 (19.1%)	832 (17.5%)	
Current smoker	249 (21.0%)	874 (18.4%)	
*Missing*	*250*	*954*	
Alcohol prior to cancer diagnosis			
Never consumed alcohol	203 (19.6)	806 (19.2)	0.94
Alcohol consumer	831 (80.4)	3,399 (80.8)	
*Missing*	*401*	*1,492*	
BMI (kg/m^2^) prior to cancer diagnosis:	1,049	4,333	
*Mean (SD)*	26.6 (5.4)	26.3 (5.1)	0.04
Comorbidity prior to cancer diagnosis			
Cerebrovascular disease	67 (4.7)	219 (3.8)	0.15
Chronic pulmonary disease	249 (17.4)	906 (15.9)	0.18
Congestive heart disease	38 (2.7)	140 (2.5)	0.72
Diabetes	81 (5.6)	274 (4.8)	0.18
Myocardial infarction	20 (1.4)	79 (1.4)	0.97
Peptic ulcer disease	35 (2.4)	145 (2.6)	0.81
Peripheral vascular disease	35 (2.4)	73 (1.3)	0.001
Rheumatological disease	62 (4.3)	187 (3.3)	0.07

### Association between receipt of prescriptions for aspirin and survival

The association between receipt of prescriptions for aspirin and cancer-specific death is shown in Table 2. There was little evidence of an association between receiving at least one prescription for low-dose aspirin after cancer diagnosis and breast cancer-specific death (adjusted OR = 1.00, 95% CI 0.71, 1.41; *P* = 0.99). There was also little evidence of dose response association between low-dose aspirin and breast cancer-specific death when the number of prescriptions, tablets or prescriptions per day was investigated. Finally, there was no significant association between receiving at least one prescription for high dose aspirin (prescribed for approximately 1% of cases and controls) and cancer-specific mortality. Similar analysis for all-cause mortality (see Table 3) showed little evidence of associations between low-dose aspirin and mortality.

**Table 2:** Post-diagnostic exposure to aspirin and odds of breast cancer-specific death in breast cancer patients

**Post-diagnostic aspirin exposure**	**Breast cancer-specific deaths n (%)**	**Controls n (%)**	**Unadjusted OR (95% CI)**	***P***	**Adjusted**^**a **^**OR (95% CI)**	***P***	**Additionally adjusted**^**b **^**for stage and grade OR (95% CI)**	***P***
No. prescriptions low dose								
0	1,173 (81.7)	4,641 (81.5)	1.00		1.00		1.00	
1 or more	262 (18.3)	1,056 (18.5)	0.98 (0.83, 1.15)	0.77	0.98 (0.81, 1.20)	0.86	1.00 (0.71, 1.41)	0.99
No. prescriptions low dose								
0	1,173 (81.7)	4,641 (81.5)	1.00		1.00		1.00	
1 to 11	117 (8.2)	525 (9.2)	0.88 (0.71, 1.09)	0.25	0.90 (0.71, 1.16)	0.42	0.80 (0.52, 1.24)	0.32
12 or more	145 (10.1)	531 (9.3)	1.08 (0.87, 1.33)	0.48	1.08 (0.84, 1.39)	0.57	1.28 (0.82, 2.01)	0.27
No. tablets low dose								
0	1,173 (81.7)	4,641 (81.5)	1.00		1.00		1.00	
1 to 365	90 (6.3)	385 (6.8)	0.93 (0.72, 1.19)	0.55	1.01 (0.77, 1.33)	0.93	0.93 (0.57, 1.52)	0.78
366 or more	172 (12.0)	671 (11.8)	1.01 (0.83, 1.22)	0.95	0.96 (0.76, 1.22)	0.76	1.05 (0.69, 1.58)	0.83
No. tablets low dose per day								
0	1,173	4,641 (81.5)	1.00		1.00		1.00	
0 to 0.5	93 (6.5)	370 (6.5)	0.99 (0.78, 1.27)	0.97	1.02 (0.78, 1.34)	0.89	0.89 (0.56, 1.42)	0.63
>0.5	169 (11.8)	686 (12.0)	0.97 (0.80, 1.17)	0.72	0.96 (0.76, 1.21)	0.73	1.10 (0.72, 1.67)	0.67
No prescriptions high dose								
0	1,415 (98.6)	5,642 (99.0)	1.00		1.00		1.00	
1 or more	20 (1.4)	55 (1.0)	1.47 (0.87, 2.46)	0.15	1.15 (0.65, 2.04)	0.62	1.11 (0.31, 4.04)	0.87

^a^Model includes chemotherapy within six months of diagnosis, radiotherapy within six months, tamoxifen (post diagnosis, during exposure period), aromatase inhibitors (post diagnosis, during exposure period), comorbidities (pre-diagnosis or during exposure period, including myocardial infarction, cerebrovascular disease, congestive heart disease, chronic pulmonary disease, peripheral vascular disease, peptic ulcer disease and diabetes), other medication exposure (post diagnosis, during exposure period, including statins, beta-blockers, ACE inhibitors, ARBs and metfomin) and smoking (pre-diagnosis, with missing included as a category).

^b^Adjusted model additionally includes stage and grade, restricted to 574 cases and 2,268 controls with available data.

**Table 3:** Post-diagnostic exposure to aspirin and odds of all-cause mortality cancer-specific death in breast cancer patients

**Post-diagnostic aspirin exposure**	**All cause deaths n (%)**	**Controls n (%)**	**Unadjusted OR (95% CI)**	***P***	**Adjusted**^**a **^**OR (95% CI)**	***P***	**Additionally adjusted**^**b **^**for stage and grade OR (95% CI)**	***P***
No. prescriptions low dose								
0	1,653 (73.2)	5,153 (76.5)	1.00		1.00		1.00	
1 or more	605 (26.8)	1,580 (23.5)	1.23 (1.09, 1.38)	0.001	1.14 (0.99, 1.31)	0.07	1.19 (0.93, 1.52)	0.17
No. prescriptions low dose								
0	1,653 (73.2)	5,153 (76.5)	1.00		1.00		1.00	
1 to 11	246 (10.9)	704 (10.5)	1.11 (0.95, 1.31)	0.19	1.04 (0.87, 1.25)	0.64	1.13 (0.83, 1.56)	0.42
12 or more	359 (15.9)	876 (13.0)	1.33 (1.15, 1.54)	<0.001	1.24 (1.04, 1.47)	0.02	1.23 (0.92, 1.66)	0.17
No. tablets low dose								
0	1,653 (73.2)	5,153 (76.5)	1.00		1.00		1.00	
1 to 365	192 (8.5)	487 (7.2)	1.25 (1.05, 1.50)	0.01	1.21 (0.99, 1.47)	0.06	1.18 (0.84, 1.67)	0.33
366 or more	413 (18.3)	1,093 (16.2)	1.21 (1.06, 1.39)	0.006	1.10 (0.94, 1.30)	0.23	1.19 (0.90, 1.59)	0.22
No. tablets (low dose) per day								
0	1,653 (73.2)	5,153 (76.5)	1.00		1.00		1.00	
0 to 0.5	225 (10.0)	590 (8.8)	1.21 (1.02, 1.44)	0.03	1.15 (0.95, 1.38)	0.16	1.22 (0.88, 1.67)	0.23
>0.5	380 (16.8)	990 (14.7)	1.24 (1.07, 1.42)	0.003	1.14 (0.97, 1.34)	0.12	1.17 (0.87, 1.57)	0.30
No prescriptions high dose								
0	2,222 (98.4)	6,660 (98.9)	1.00		1.00		1.00	
1 or more	36 (1.6)	69 (1.0)	1.48 (0.99, 2.21)	0.05	1.24 (0.81, 1.89)	0.32	0.94 (0.48, 1.84)	0.86

^a^Model includes chemotherapy within six months of diagnosis, radiotherapy within six months, tamoxifen (post diagnosis, during exposure period), aromatase inhibitors (post diagnosis, during exposure period), comorbidities (pre-diagnosis or during exposure period, including myocardial infarction, cerebrovascular disease, congestive heart disease, chronic pulmonary disease, peripheral vascular disease, peptic ulcer disease and diabetes), other medication exposure (post diagnosis, during exposure period, including statins, beta-blockers, ACE inhibitors, ARBs and metfomin), and smoking (pre-diagnosis, with missing included as a category).

^b^Adjusted model additionally includes stage and grade, restricted to 888 cases and 2,628 controls with available data.

CI, Confidence interval; OR, Odds ratio.

### Sensitivity analyses

Sensitivity analyses for the association between low-dose aspirin exposure and cancer-specific death are shown in Table 4. Findings were little altered when the exposure period was varied or when aspirin prescriptions prior to breast cancer diagnosis were investigated. There was also no evidence of a difference in the association between low-dose aspirin exposure after cancer diagnosis and breast cancer-specific death when stratifying by aspirin exposure prior to diagnosis, or when stratifying by stage or when restricted to patients receiving hormone therapy (within six months of diagnosis). No association was seen in breast cancer patients diagnosed with early stage disease (stage 1 and 2). Defining breast cancer-specific death based upon a breast cancer code for any cause of death, not necessarily the main cause, also had little impact on the results (OR = 1.05, 95% CI 0.91, 1.20). Restricting the adjusted analysis to cancer registries where stage recording was 85% complete also had little impact on the results. Repeating the analysis in the entire breast cancer cohort using a Cox proportional hazards model with a time varying covariate produced similar estimates (hazard ratio (HR) = 1.06, 95% CI 0.92, 1.22) and additionally accounting for competing risks of deaths from other causes had little impact (sub-distribution HR = 1.04, 95% CI 0.81, 1.08).

**Table 4:** Sensitivity analysis for association between low dose aspirin exposure and breast cancer-specific death

**Comparison**^**a**^	**Cancer-specific deaths**	**Controls**	**OR (95% CI) low dose aspirin exposed vs. non-exposed**	***P***	**OR (95% CI) 1 to 11 low dose aspirin prescriptions vs. none**	***P***	**OR (95% CI) 12 or more low dose aspirin prescriptions vs. none**	***P***
Main analysis: Diagnosis to six months prior to death	1,435	5,697	0.98 (0.83, 1.15)	0.77	0.88 (0.71, 1.09)	0.25	1.08 (0.87, 1.33)	0.48
Post diagnostic aspirin in patients with no pre-diagnostic aspirin prescriptions^b^	1,125	4,457	0.82 (0.64, 1.04)	0.10	0.81 (0.59, 1.10)	0.17	0.84 (0.59, 1.18)	0.31
Post diagnostic aspirin in patients with pre-diagnostic aspirin prescriptions^b^	142	515	0.95 (0.52, 1.71)	0.86	0.82 (0.43, 1.56)	0.55	1.08 (0.57, 2.03)	0.81
Pre-diagnostic low dose aspirin prescriptions^c^	1,559	6,186	0.99 (0.83, 1.18)	0.93	1.01 (0.83, 1.22)	0.93	0.94 (0.67, 1.32)	0.71
Diagnosis to one year prior to death^d^	1,272	5,048	1.06 (0.89, 1.26)	0.51	1.01 (0.80, 1.27)	0.95	1.11 (0.89, 1.40)	0.36
Diagnosis to two years prior to death^e^	959	3,797	0.89 (0.72, 1.10)	0.28	0.62 (0.46, 0.85)	0.003	1.22 (0.93, 1.60)	0.15
One year after diagnosis to six months prior to death^f^	1,099	4,356	0.93 (0.77, 1.12)	0.43	0.84 (0.65, 1.09)	0.20	1.01 (0.79, 1.29)	0.94
Stage 1 and 2 breast cancer patients only	469	1,856	1.09 (0.82, 1.46)	0.54	0.83 (0.55, 1.24)	0.36	1.41 (0.98, 2.04)	0.07
Stage 3 and 4 breast cancer patients only	161	478	1.13 (0.68, 1.86)	0.64	1.24 (0.66, 2.33)	0.50	0.98 (0.45, 2.12)	0.96
Restricted to patients receiving prescriptions for hormone therapy (in first six months)	875	3,466	1.08 (0.90, 1.30)	0.43	0.93 (0.72, 1.20)	0.55	1.23 (0.97, 1.56)	0.08
Patients with a recorded stage from cancer registries with high rates of stage recording (adjusted for stage)^g^	487	1,911	0.92 (0.67, 1.27)	0.62	0.95 (0.62, 1.45)	0.81	0.90 (0.59, 1.37)	0.62
Including breast cancer-specific deaths where breast cancer is recorded as any cause of death	1,786	7,090	1.05 (0.91, 1.20)	0.53	1.01 (0.84, 1.21)	0.95	1.09 (0.91, 1.30)	0.37
Time varying covariate analysis^h^	1,440	8,374	1.06 (0.92, 1.22)	0.45	0.98 (0.81, 1.20)	0.88	1.13 (0.94, 1.35)	0.20
Time varying covariate analysis^i^	1,440	8,374	1.04 (0.90, 1.20)	0.60	0.96 (0.79, 1.67)	0.67	1.12 (0.93, 1.35)	0.22

^a^All sensitivity analyses refer to low dose aspirin prescriptions in the time period from breast cancer diagnosis to six months before death, and are adjusted for matching criteria only, unless otherwise stated.

^b^Pre-diagnostic low dose aspirin prescriptions in one year prior to breast cancer diagnosis, restricted to individuals with one year of medication records prior to diagnosis.

^c^Pre-diagnostic low dose aspirin prescriptions in one year prior to breast cancer diagnosis.

^d^Restricted to individuals with at least 1.5 years of follow-up so relevant exposure period is at least a duration of six months.

^e^Restricted to individuals with at least 2.5 years of follow-up so relevant exposure period is at least a duration of six months.

^f^Restricted to individuals with at least two years of follow-up so relevant exposure period is at least a duration of six months.

^g^Stage missing for 15% of individuals and breast cancer patients included from the Northern and Yorkshire Cancer Registry and Information Service, the Trent Cancer Registry, the Eastern Cancer Registration and Information Centre, the Oxford Cancer Intelligence Unit and the West Midlands Cancer Intelligence.

^h^Reported estimates are hazard ratios and 95% confidence intervals (CIs), adjusted for age and year of breast cancer diagnosis.

^i^Reported estimates are sub-distribution hazards ratios and 95% CIs, adjusted for competing risks of death, age and year of breast cancer diagnosis.

## Discussion

In this large UK population-based cohort of breast cancer patients, there was little evidence of an association between post-diagnostic exposure to low-dose aspirin (determined from prescription records) and breast cancer-specific survival. This lack of association was seen irrespective of whether the breast cancer patient had received prescriptions for aspirin pre-diagnostically, and pre-diagnostic exposure to low-dose aspirin was not associated with cancer-specific mortality. No association was seen in breast cancer patients diagnosed with early stage disease or in patients who received prescriptions for hormone therapy (presumed to be oestrogen receptor positive cases).

Data from two US studies, the Nurses’ Health Study [4] and the Iowa Women’s Health Study [3], indicate a substantial (approximately 50%) reduction in risk of cancer progression in early stage breast cancer patients using aspirin post-diagnostically, although another US study (the LACE study) [6] did not observe any risk reduction in aspirin users and a study of breast cancer patients recruited in New York state showed no association between recent pre-diagnostic aspirin exposure and breast cancer survival [20]. Information on aspirin dosage was not available in these studies but high dose aspirin is more commonly used in the US than in the UK [21]. Indications for aspirin use were explored in the Nurses’ Health Study; 35% of users took aspirin (most likely low-dose) for protection against cardiovascular disease while 37% used aspirin (presumably high dose) for musculoskeletal pain, headache or menstrual pain (data on other indications were not provided). Therefore, the reduction in risk of cancer progression seen in the US breast cancer studies may stem from PTSG-2 inhibition by high dose aspirin rather than platelet inhibition from use of low-dose aspirin. Similar reductions in risk observed for non-aspirin NSAIDs [3,6], such as ibuprofen, suggest that this is the case. Use of aspirin at sufficient doses to cause PTSG-2 inhibition may be required to prevent breast cancer progression and although platelets appear to play an important role in cancer progression [10], especially metastasis formation, our data do not suggest a role for platelet inhibition by aspirin in the prevention of breast cancer progression, even in patients diagnosed with early stage disease.

This study has several strengths. It included a population representative sample of breast cancer patients and is the largest study in the field to date. Prior to this work, the largest relevant study included 4,461 breast cancer patients, in whom there were 292 breast cancer deaths [4], whereas this study included 9,817 breast cancer patients and 1,443 cancer-specific deaths. We also investigated receipt of aspirin prescriptions in the most relevant exposure period; that is, following diagnosis when treatments may be implemented to prevent cancer progression. Thus far, only two other studies have investigated post-diagnostic exposure to aspirin [4,6]. Follow-up in our study was for a reasonably long period in order for sufficient events to occur and post-diagnostic exposure to low-dose aspirin was common, allowing adequate precision around the risk estimates. In contrast, high dose aspirin was infrequently prescribed in our cohort and consequently risk estimates were imprecisely estimated. The linkage with NCDR and ONS allowed robust verification of cancer diagnosis and death data. The use of GP prescribed drug information eliminated the potential for recall bias incurred by self-report, and allowed temporal associations to be explored [22]. The drug data reflect GP prescriptions, not drugs dispensed or actually consumed, and compliance cannot be assumed. The potential non-compliance of cases and controls with prescribed aspirin therapy is a limitation of this study, and may have contributed to the null findings. However, we observed null associations in analyses of patients who received multiple aspirin prescriptions or those with at least one year’s prescription and so do not believe that non-compliance greatly impacted upon the results shown. Over-the-counter usage of aspirin in cases and controls must be considered as our inability to capture such use may have also contributed to the null findings. However, the amount of over-the-counter use is likely to be low as a previous General Practice Research Database (GPRD) study [23] comparing prescription records to patient self–report noted that the majority of chronic aspirin therapy was captured by prescription records and another estimated that 70% to 80% of aspirin use in the age-group we investigated was prescription based [24]. Furthermore, previous methodological studies have shown that even with high levels of over the counter usage (much higher than is likely to have occurred in our study) valid treatment effects can be obtained using prescription databases [25].

We were unable to stratify analysis by tumour molecular subtypes or by tumour markers related to metastasis risk [26]. No association was observed in patients receiving prescriptions for hormone therapy (a proxy for oestrogen receptor positivity) and previous investigators have not seen differences in the association between aspirin exposure and breast cancer progression by hormone receptor status [4,6]. However, further studies should examine the association between aspirin use and breast cancer progression according to relevant tumour molecular characteristics. A further limitation was our inability to study breast cancer metastasis or recurrence as an outcome, since this information is not routinely collected in the NCDR. Although it is possible that some deaths have been incorrectly misclassified when determining cancer-specific mortality, simulations from a recent methodological study indicate that in comparative studies in which differential misclassification between groups is unlikely, like ours, such misclassification is unlikely to impact on effect estimates [27]. We did not have complete data on potentially important confounders such as BMI and smoking and there also remains the possibility of residual confounding from unidentified confounders; however, previous reports did not show evidence of substantial confounding of the association between aspirin and breast cancer progression by factors, such BMI, physical activity, smoking, race and so on [3,4,6]. Although stage data were incomplete, it seems unlikely that stage confounded the main finding as adjustments for stage in the subset of cancer registries where stage data were 85% complete had little influence on risk estimates. Confounding by indication is often problematic within pharmaco-epidemiological studies [28] and this may be expected in an examination of the association between use of high dose aspirin or non-aspirin NSAIDs and cancer progression as cancer patients may use these drugs for pain relief; however, indication for use is unlikely to confound the association between exposure to low-dose aspirin and cancer progression as aspirin at these doses is not used for the management of pain.

Regular use of aspirin has increased substantially in recent years, for example, the proportion of the US population taking aspirin at least three times a week over a three-month period increased from 19.3% to 27.4% (1.4-fold increase) between 2005 and 2010 [29]. Even higher increases in use were seen for cancer patients, including breast cancer patients in whom regular use increased from 21.3% to 37.9% (1.8-fold increase). Reasons for prescribing aspirin in the cancer patients was not available nor was timing of prescribing with respect to the date of cancer diagnosis so it is unclear whether aspirin was prescribed in an attempt to prevent cancer progression. Current evidence does not support aspirin use for such an indication, but this is an active area of research. At least one trial of aspirin therapy, including low-dose therapy, in cancer patients, the Add-Aspirin Trial (includes breast cancer patients) is underway but it will not report until 2020 at the earliest [30]. In the interim, data from robust observational studies are informative.

## Conclusion

In our study, there was little evidence of an association between receipt of post-diagnostic prescriptions for low-dose aspirin and breast cancer-specific death. However, information was not available on medication compliance or over-the-counter use of aspirin, which may have contributed to the null findings.

## Abbreviations

ACE: Angiotensin converting enzyme; ARB: Angiotensin receptor blocker; BMI: Body mass index; BNF: British national formulary; CI: Confidence interval; CPRD: Clinical practice research datalink; GPRD: General practice research database; GP: General practitioner; HR: Hazard ratio; HRT: Hormone replacement therapy; ICD: International classification of diseases; LACE study: Life after cancer epidemiology study; MREC: Multicentre research ethics committee; NCDR: National cancer data repository; NSAIDs: Non-steroidal anti-inflammatory drugs; ONS: Office of national statistics; OR: Odds ratio; PTSG-2: Prostaglandin endoperoxide synthase-2; UK: United Kingdom; US: United States.

## Competing interests

The authors declare that they have no competing interests.

## Authors’ contributions

CRC, LJM and CMH conceived and designed the study. CRC, LJM, DGP and CMH were involved in data acquisition. CRC and JAC conducted statistical analysis. CRC, LJM, DGP, JAC and CMH were involved in interpretation of the data. LJM drafted the manuscript. All co-authors revised the manuscript and have given final approval for publication. LJM takes final responsibility.
